# A *Bacillus velezensis* strain isolated from oats with disease-preventing and growth-promoting properties

**DOI:** 10.1038/s41598-024-63756-8

**Published:** 2024-06-05

**Authors:** Chao Cheng, Shaofeng Su, Suling Bo, Chengzhong Zheng, Chunfang Liu, Linchong Zhang, Songhe Xu, Xiaoyun Wang, Pengfei Gao, Kongxi Fan, Yiwei He, Di Zhou, Yanchun Gong, Gang Zhong, Zhiguo Liu

**Affiliations:** 1 School of Life Science and Technology, Jining Normal University, Ulanqab, 012000 China; 2 Inner Mongolia Academy of Agriculture and Husbandry Science, Key Laboratory of Black Soil Protection and Utilization, Ministry of Agriculture and Rural Affairs, Hohhot, 010000 China; 3 College of Computer Information, Inner Mongolia Medical University, Hohhot, 010000 China; 4 Ulanqab Institute for Agricultural and Forestry Science, Ulanqab, 012000 China; 5 Ulanqab Center for Disease Control and Prevention, Ulanqab, 012000 China; 6 Jinyu Baoling Biological Drugs Co., LTD, Hohhot, 010000 China; 7 Vocational and Technical College of Ulanqab, Ulanqab, 012000 China; 8 Inner Mongolia Agricultural University, Hohhot, 010000 China; 9 The Second Affiliated Hospital of Inner Mongolia Medical University, Hohhot, 010000 China; 10 Agriculture and Animal Husbandry Technology Promotion Center of Inner Mongolia, Hohhot, 010000 China; 11 Chinese Center for Disease Control and Prevention, National Institute for Communicable Disease Control and Prevention, Beijing, 100000 China

**Keywords:** *Bacillus velezensis*, Antibacterial activity, Complete genome, Comparative genome, Microbiology, Plant sciences

## Abstract

Endophytes have been shown to promote plant growth and health. In the present study, a *Bacillus velezensis* CH1 (CH1) strain was isolated and identified from high-quality oats, which was capable of producing indole-3-acetic acid (IAA) and strong biofilms, and capabilities in the nitrogen-fixing and iron carriers. CH1 has a 3920 kb chromosome with 47.3% GC content and 3776 code genes. Compared genome analysis showed that the largest proportion of the COG database was metabolism-related (44.79%), and 1135 out of 1508 genes were associated with the function “biosynthesis, transport, and catabolism of secondary metabolites.” Furthermore, thirteen gene clusters had been identified in CH1, which were responsible for the synthesis of fifteen secondary metabolites that exhibit antifungal and antibacterial properties. Additionally, the strain harbors genes involved in plant growth promotion, such as seven putative genes for IAA production, spermidine and polyamine synthase genes, along with multiple membrane-associated genes. The enrichment of these functions was strong evidence of the antimicrobial properties of strain CH1, which has the potential to be a biofertilizer for promoting oat growth and disease resistance.

## Introduction

Plants have a high diversity of microbial communities, which are essential for plant health. Beneficial microorganisms can promote plant growth, alleviate plant stress, and defend against plant pathogens through various mechanisms of interaction with plants^1^. *Bacillus velezensis* is regarded as a new beneficial microorganism found in plants and root soil in recent years that is rich in metabolites and exhibits broad-spectrum antibacterial activity and a strong antibacterial ability, which can promote plant growth and enhance salt stress tolerance^2,3^. *B. velezensis* has been shown to produce abundant cytokinins and to promote plant growth using the plant to produce volatile organic compounds, such as IAA^4^. In addition, *B. velezensis* has also been shown to have a nitrogen-fixing reductase (60 kDa), which has potential biological control characteristics against the host rhizosphere pest *Spodoptera striata*^5^. Moreover, *B. velezensis* was also found to highly express the antimicrobial peptide gene, which might be the reason for the biocontrol of the strain^5^. Furthermore, *B. velezensis* reduced the number of threadworm eggs in tomato roots and soil and inhibited the incidence of tomato plants^6^. *B. velezensis* AH2 demonstrated resistance to *Saprophytes, Phytophthora, Sclerotinia, Mycelium, Penicillium*, and *Alternaria*^7^, and strong inhibition of plant diseases such as rice blast, rice sheath blight, pepper anthracnose, tomato gray mold, wheat root rot, and barley powdery mildew^8^.

As a type of grain, oats are not only one of the main food sources but also an important feed for livestock globally. The main planting areas of oats are mountainous, plateaus, and high cold areas in China. The Ulanqab region of Inner Mongolia is one of the main oat-producing areas in China. Its planting area and output both rank first among prefectural-level cities in China, and it is an important local food crop. *B. velezensis* has been found in many plants with specific functions, but it is not well-studied in oats. Therefore, in the present study, we screened, isolated, purified, and identified *B. velezensis* from high-quality oats and characterized its phenotypic features by applying a series of biochemical procedures. We then conducted a whole genome comparison analysis of *B. velezensis* to explore the molecular mechanism by which *B. velezensis* promoted the growth and pest control of oats from a molecular perspective.

## Results

### Identification and biochemical features of the CH1 strain

**Figure 1:**
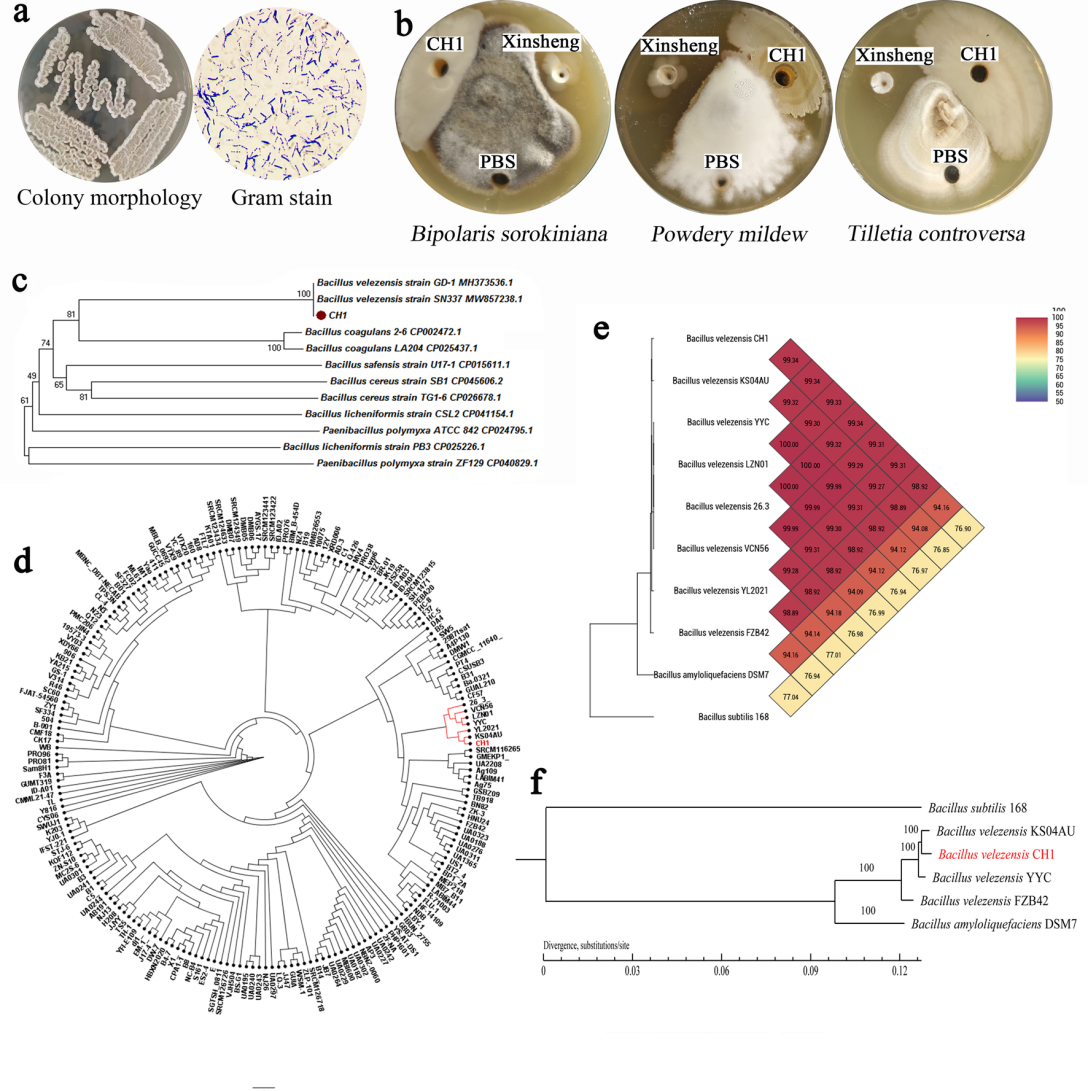
Screening and Phylogenetic Analysis of Strain CH1. (**a**) Morphological identification of CH1. (**b**) The antimicrobial activity of the CH1. (**c**) The result of the evolutionary tree of CH1. (**d**) Phylogenetic tree depicting the relationship between CH1 and 201 reference *B. velezensis* strains. (**e**) OrthoANI values were calculated using the genomic sequences between strains. (**f**)The phylogenetic tree is drawn from the core genomes of CH1, *Bacillus velezensis* KS04AU (KS04AU), *Bacillus velezensis* YYC (YYC), *Bacillus velezensis* FZB42 (FZB42), *Bacillus velezensis* DSM7 (DSM7) and *Bacillus velezensis* 168 (168). Bootstrap values are indicated in % of repetitions.

CH1 has a protruding, irregularly round shape with concentric circles in the center and white mucus inside (Fig. 1a). It is a Gram-positive bacillus (Fig. 1a) and exhibits a series of beneficial microorganism features such as aerobic or facultative anaerobic respiration, motion, catalase and VP positive, gelatin liquefaction positive, and indole negative, and is able to ferment glucose, arabinose, xylose, mannose, and starch. The 16s RNA sequence analysis showed that there was a 100% homology similarity of the CH1 16s DNA sequence to *Bacillus velezensis* strain GD-1 (MH373536.1) and *Bacillus velezensis* strain SN337 (MW857238.1) (Fig. 1c). CH1 grew weakly in an agar medium with ACC as the only nitrogen source (Supplementary Fig. S1) and produced a blue color in the Nfb solid medium compared with the blank medium (Supplementary Fig. S2). The present study confirmed that CH1 did not demonstrate phosphate solubility in NBRIP solid medium (Supplementary Fig. S3), but did produce iron carriers (Supplementary Fig. S4). In addition, CH1 had a significant capacity for generating IAA (Table 1) and biofilms (Table 2) and had a strong inhibitory effect on *Bipolaris sorokiniana*, *Powdery mildew*, and *Tilletia controversa* (Table 3, Fig. 1b).

**Table 1:** The results of determination of IAA (n = 3, X ± SD).

	Blank control (ng/mL)	Positive control (ng/mL)	CH1 (ng/mL)
Concentration	2.2 ± 0.0039	2.1 ± 0.0056	2.63 ± 0.0023*

The culture medium TSB of uninoculated bacteria was used as blank control to remove the influence of the medium on the results, and *Bacillus subtilis* was the positive control group. The ability of CH1 to produce IAA was significantly higher than that of the blank control group and the positive control group (*P* < 0.05). This showed that CH1 had a significant capacity for generating IAA. Each value represents the mean value standard deviation (SD) from three trials under the same experimental conditions. *The significant difference compared with blank control (*P* < 0.05), **Indicates the extremely significant difference compared with blank control (*P* < 0.01).

**Table 2:** Results of CH1 biofilm formation (n = 3, X ± SD).

	Blank control (CFU/mL)	Positive control (CFU/mL)	CH1 (CFU/mL)
Concentration	(0.42 ± 0.002) × 10^9^	(0.66 ± 0.08) × 10^9^	(2.78 ± 1.76) × 10^9^**

The culture medium TSB of uninoculated bacteria was used as blank control to remove the influence of the medium on the results, *Bacillus subtilis* was the positive control group. The ability of CH1 to produce biofilm was extremely significant higher than that of the blank control group and positive control group (*P* < 0.01). Each value represents the mean value standard deviation (SD) from three trials under the same experimental conditions. *Indicates the significant difference compared with blank control (*P* < 0.05), **Indicates the extremely significant difference compared with blank control (*P* < 0.01).

**Table 3:** Antimicrobial activity analysis of CH1 (n = 3, X ± SD).

	Bipolaris sorokiniana (mm)	Powdery mildew (mm)	Tilletia controversa (mm)
CH1	12 ± 0.71**^#^	13 ± 0.15**^#^	12 ± 0.22**
Positive control	9 ± 0.13**	16 ± 0.21**	11 ± 0.44**
Blank control	0	0	0

Phosphate buffer (PBS) was used as the blank control group, and XinSheng, a product on the market for inhibiting *Bipolaris sorokiniana, Powdery mildew,* and *Tilletia controversa*, was used as the positive control group in this experiment. The inhibition of CH1 and positive control on pathogens were extremely significant different from that of the blank control group (*P* < 0.01), and CH1 decreased significantly to *Powdery mildew* compared with positive control group (*P* < 0.05), CH1 increased significantly in *Bipolaris sorokiniana* compared with the positive control group (*P* < 0.05), CH1 had no significant difference in *Tilletia controversa* compared with positive control group (*P* > 0.05). Each value represents the mean value standard deviation (SD) from three trials under the same experimental conditions. * The significant difference compared with blank control (*P* < 0.05), ** The extremely significant difference compared with blank control (*P* < 0.01). ^#^ The significant difference compared with the positive control group (*P* < 0.05), ^##^ The extreme significant difference compared with the positive control group (*P* < 0.01).

### Whole-genome sequencing profiling and COG functional annotation of strain CH1

**Figure 2:**
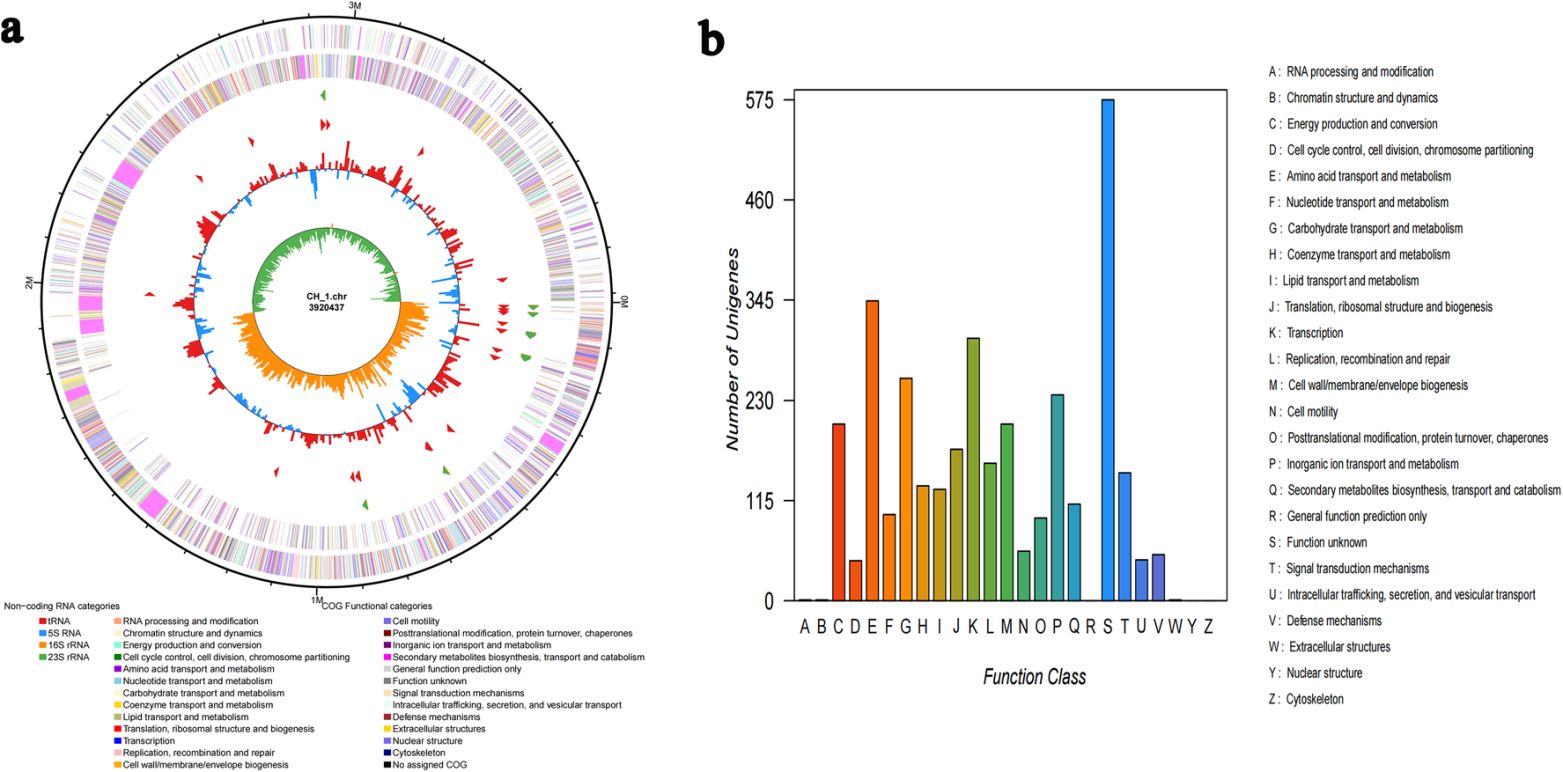
Whole Genome Structure and Functional Analysis of Strain CH1. (**a**) Circle diagram of CH1 genome. (**b**) The statistical results of the Clusters of Orthologous Groups of proteins (COG) functional classification of genomic protein.

A total of 494,097 reads were sequenced with an average length of 882 bp. The CH1 strain was assembled into a round chromosome with a size of 392,0437 bp, and the guanine-cytosine (GC) content of the total genome was 47.3% (Table 4). There are 3776 code genes in the genome of the CH1 strain, 27 in rRNA, 86 in tRNA, and 93 in ncRNA. The genome circle map was constructed based on the COG database annotation results (Fig. 2a). Metabolism accounted for the largest proportion of annotations (44.79%), and this category was considered to be related to predicted secondary metabolic activity proteins. Among them, 1508 genes were annotated into 1135 COGs, which were divided into eight types of functions (Fig. 2b). Proteins related to “secondary metabolite biosynthesis, transport, and catabolism” accounted for 7.36%. The enrichment of these functions was strong evidence for the antibacterial properties of strain CH1.

**Table 4:** Statistical table of CH1 genome components.

Feature	Value
Gene number	3776
Gene total length	3,500,379
Gene average length	927
Gene density, genes per kb	0.963
GC content in gene regions (%)	47.3%
Gene/Genome (%)	89.3%
tRNA genes	86
rRNA genes	27
ncRNA	93
5S rRNA	9
16S rRNA	9
23S rRNA	9

A genome-based phylogenetic tree was constructed between strain CH1 and 201 *B. velezensis* strains in the NCBI genome database. The dendrogram showed that strain CH1 was closely related to strains KS04AU (CP092750.1) and *Bacillus velezensis* YL2021 (YL2021) (CP120641.1) and clustered with YYC (CP075055.1) and *Bacillus velezensis* LZN01 (LZN01) (CP081488.1) (Fig. 1d). It has been reported that the Ortho ANI value of 95% (corresponding to 70% DDH) can be used as a cut-off point for bacterial species classificatio^9,10^. Using the genome sequences of strain CH1 and six Bacillus genera, OrthoANI values were calculated. Strain CH1 showed the highest similarity with KS04AU, YYC and *Bacillus velezensis* 26.3 (26.3) (99.34%), followed by LZN01 (99.33%), *Bacillus velezensis* VCN56 (VCN56) (99.31%), YL2021 (99.31%), and FZB42 (98.92%) (Fig. 1e). Additionally, the dDDH values between strain CH1 and YYC, KS04AU, LZN01, 26.3, VCN56, YL2021, and FZB42 ranged from 95.70% to 97.90% (Supplementary Table S1). Based on the above results, six strains of *B. velezensis* with the closest affinity were selected to construct a phylogenetic tree based on the full-length 16S rRNA gene with the target strains, and the results showed that strain CH1 was the closest affinity to YYC. (Supplementary Fig. S5).Taking it all together, we selected KS04AU and YYC as well as the *Bacillus* model strains for comparative analysis.

### Comparative genomics analysis of CH1

**Figure 3:**
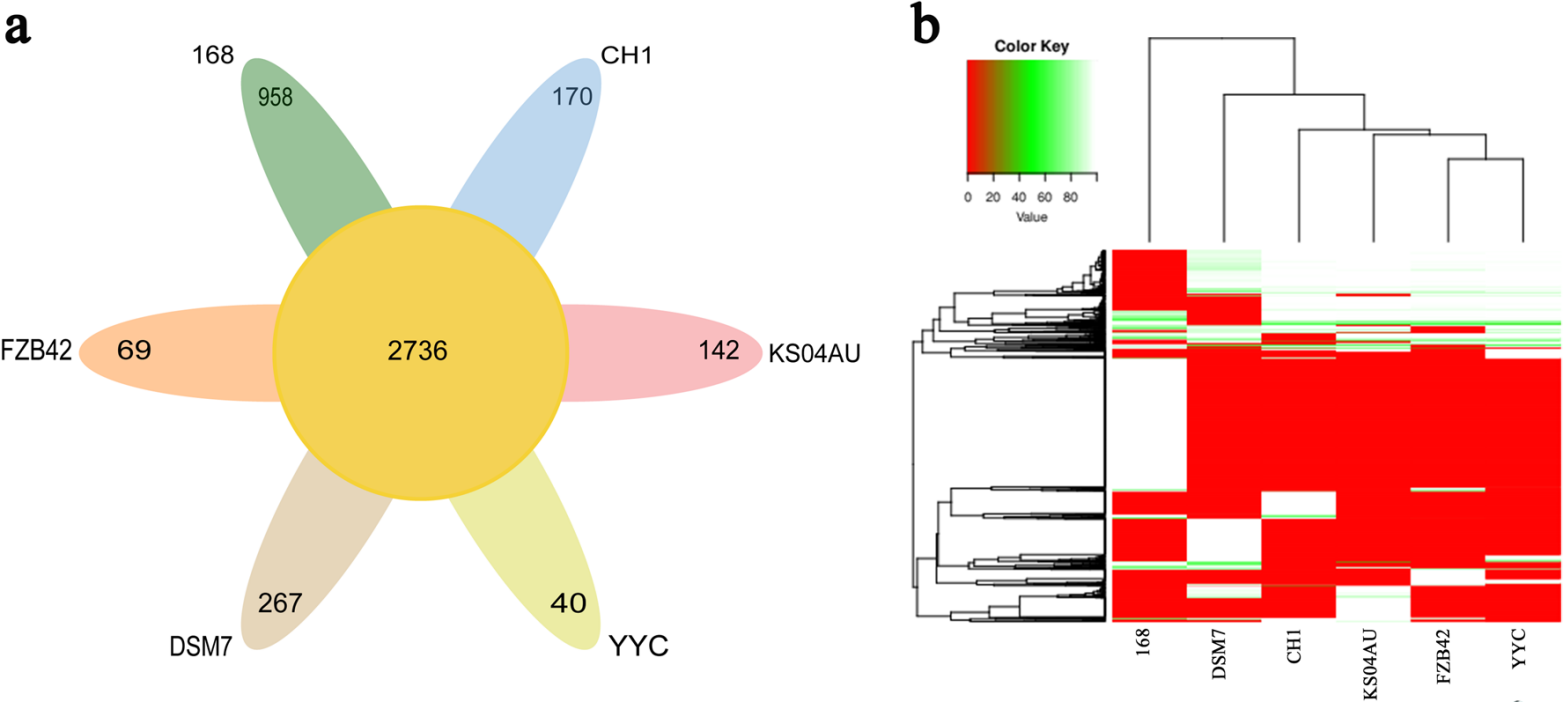
Comparative genomic analysis of strain CH1. (**a**) CorePan genome analysis of bacteria strains. (**b**) Venn diagram showing the distribution of orthologous gene families in the genomes of CH1, KS04AU, YYC, FZB42, DSM7 and 168. This analysis exploits all CDS of the genomes and is not restricted to the core genome. The number above each part represents the number of gene families, while the number below (in parentheses) represents the number of genes.

**Figure 4:**
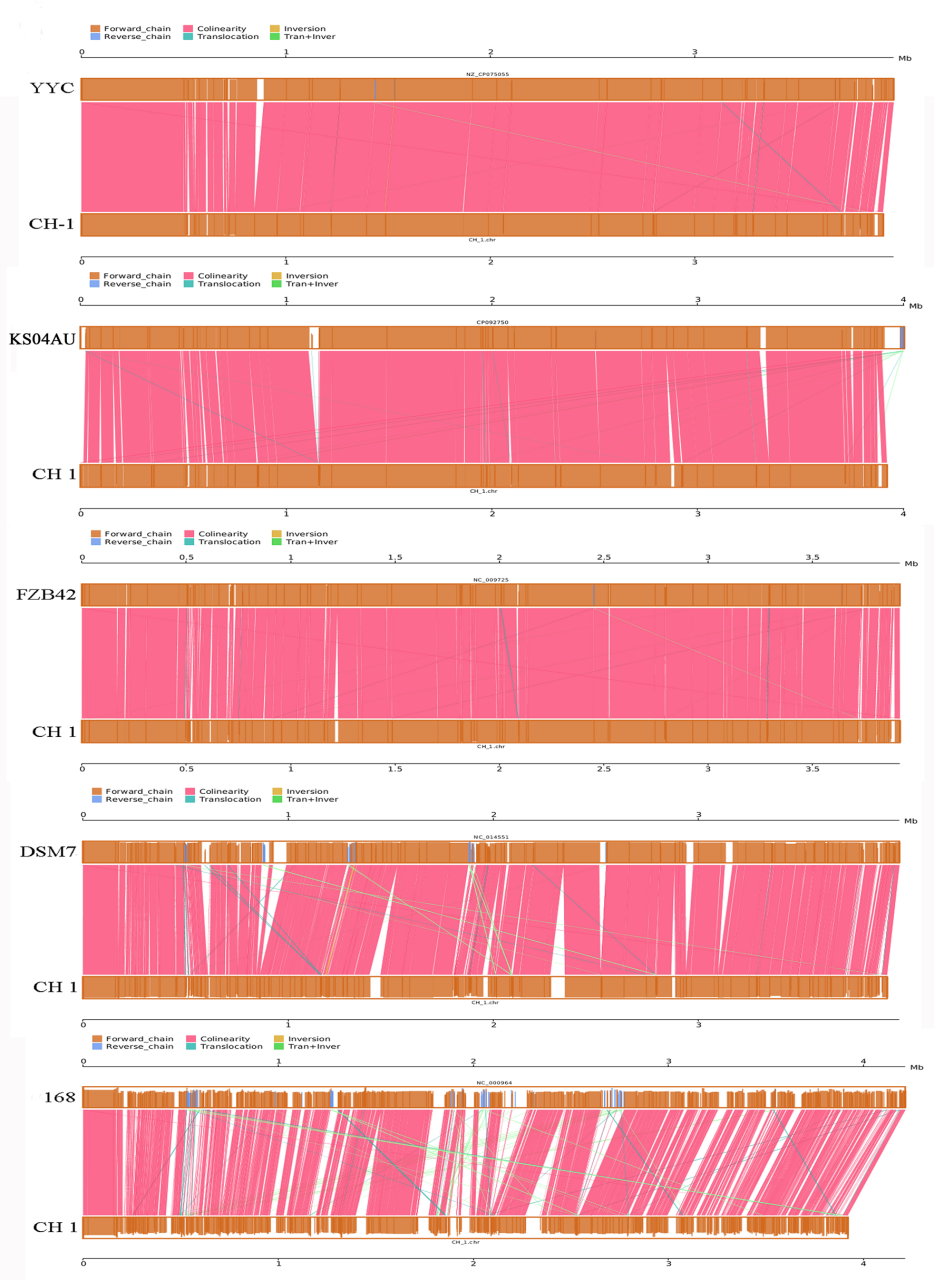
Global alignment of CH1 and *Bacillus* spp. chromosomes. Indels are depicted by gray rectangles. Dark blue indicates a sequence with synteny, red indicates forward collinearity and light blue indicates negative collinearity.

Bases from the core and pan-gene cluster analysis showed that strain CH1 formed a close genetic relationship with strain KS04AU and had obvious branching with four other strains:YYC, FZB42, DSM7 and 168 (Fig. 1f). Core- and pan-genome analysis showed that the number of non-redundant pan-genes between CH1 and the five reference strains was 4382, among which the number of shared core genes was 2736 and the number of non-shared dispensable genes was 1646. The number of specific genes in CH1 accounted for 3.9% of the total number of genes, and accounting for 3.2% and 0.9% of the total number of genes in strain KS04AU and strain YYC, respectively (Fig. 3a,b). These data suggested that the number of specific genes held by CH1 was relatively large and was scattered in the genome, while the function of most specific genes was unknown. These data demonstrated that strain CH1 was potentially a newly discovered antifungal strain. The genome collinearity analysis showed that the CH1 has a high genome similar to strain YYC (96.49%), KS04AU (95.65%) and FZB42 (95.63%) , followed by the *Bacillus amyloliquefaciens* DSM7 (DSM7) (87.95%), and 168 (67.31%) (Fig. 4). Among all the sequenced genomes, CH1 showed the highest homology with YYC, KS04AU and FZB42 indicating that they had a close relationship at a genomic scale.

### Prediction of CH1 genome-wide secondary metabolites

The CH1 strain encoded a total of 13 secondary metabolite synthesis gene clusters, among which eight gene clusters were identified with complete similarity or high similarity, and another three gene clusters could not be identified (Table 5). The secondary metabolites encoded by the CH1 strain included five non-ribosomal peptide synthases (NRPSs), four transAT-PKSs, two terpenes, two T3PKSs, one Lanthipeptide class III, and one PKS-like. Most of these were related to the production of the NRPS and PKS systems. Cluster 13 is a unique metabolite pathway, which is associated with metabolite and synthesis of the bacilysin. CH1 was found to produce bacinapeptin, surfactin, fengycin, bacillibactin, bacillaene, macolactin, difficidin, bacilysin, locillomycin, and other peptide glycans and polyketosaccharide resistance compounds. The synthesis of these secondary metabolites is one of the main mechanisms of disease prevention.

**Table 5:** Gene clusters for the synthesis of secondary metabolites in CH1, KS04AU, YYC, FZB42, DSM7 and 168.

Cluster	Gene cluster location				Presence ( +) or absence ( −)				
	Type	Size (nt)	Compound	Similarity (%)	KS04AU	YYC	FZB42	DSM7	168
1	Lanthipeptide-class-iii	22,616	Andalusicin A/Andalusicin B	100	−	+	−	−	−
2	NRPS	64,861	Surfactin	95	+	+	+	+	+
3	PKS-like	41,245	Butirosin A /Butirosin B	7	+	+	+	+	−
4	Terpene	17,171	Unknown	-	+	+	+	+	+
5	TransAT-PKS	87,819	Macrolactin H	100	+	+	+	−	−
6	TransAT-PKS, T3PKS, NRPS	100,225	Bacillaene	100	+	+	+	+	+
7	NRPS, TransAT-PKS, Betalactone	134,191	Fengycin	100	+	+	+	+	+
8	Terpene	21,884	Unknown	−	+	+	+	+	+
9	T3PKS	41,107	Unknown	−	+	+	+	+	−
10	TransAT-PKS	93,793	Difficidin	100	+	+	+	−	−
11	NRPS	50,235	Bacillothiazol A ~ N	100	+	+	+	+	+
12	NRP-metallophore, NRPS, RiPP-like	51,794	Bacillibactin	100	−	+	+	+	−
13	Other	41,419	Bacilysin	100	+	+	+	+	+

**Figure 5:**
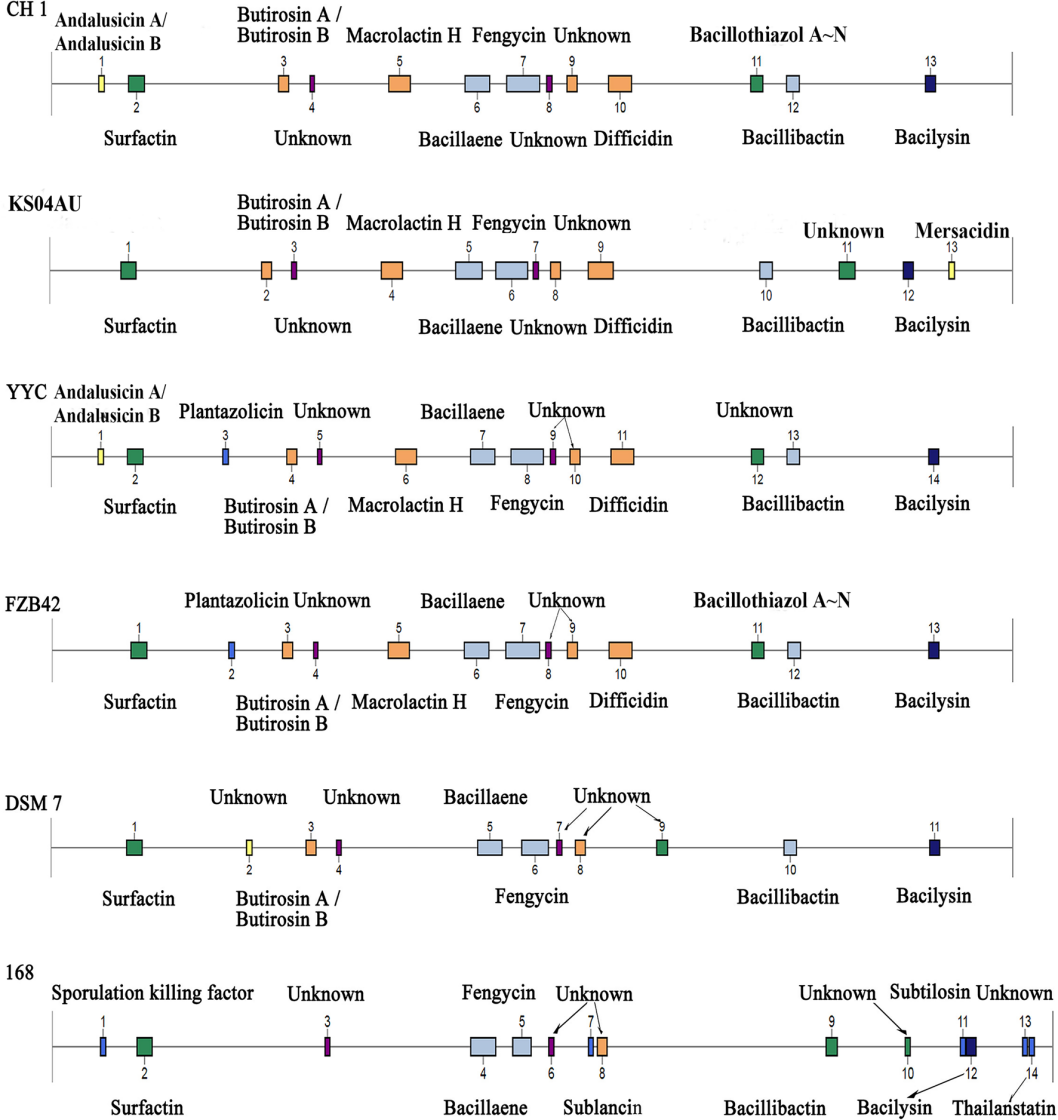
Comparative analysis of secondary metabolic gene clusters. Comparative analysis of the location and products of secondary metabolite gene clusters in CH1, KS04AU, YYC, FZB42, DSM7 and 168.

By comparing the gene clusters of CH1 with those of five other strains of *Bacillus* (KS04AU, YYC, FZB42, DSM7, and 168), it was found that six gene clusters (2, 4, 6–8, and 13) were conserved among the six strains. Two clusters (3 and 9) were conserved among CH1, KS04AU, YYC, and FZB42; two clusters (5 and 10) were conserved among CH1, KS04AU, YYC, and FZB42; and one cluster (1) was conserved between CH1 and YYC (Table 5), which encodes andalusicin. Based on comparative genomics research, we identified three clusters (4, 8, and 9) encoding terpenes and T3PKs, which were not described as previously reported due to the limited similarity of the compounds deposited in the Minimal Information on Biosynthesis Genes in Clusters (MIBiG) database (Fig. 5). Although the core biosynthetic genes of the four strains CH1, KS04AU, YYC, and FZB42 are very similar, they have different genetic structures. In the surfactin cluster, *xy02* genes were only present in CH1, *ycxC* and *ycxD* genes were lacking in DSM7, and the *yx01* gene was lost in 168. The Fengycin cluster in DSM7 lacked *fenB*, *fenC*, and *fenD*, and the *bmyC*, *bmyB*, *bmyA*, *ituA*, *ituB*, *ituD*, *bmyD*, *YxjF*, *scoB*, *scoA*, and *YxjC* genes were missing in 168 (Supplementary Table S2).

### Genetic basis for the biological activity of CH1

#### Antimicrobial activity of CH1

The CH1 genome was found to encode a variety of antimicrobial active compounds by genetic comparison of secondary metabolite gene clusters with the MIBiG database (Table 6). We selected the ones with more than 80% homology for analysis. Bacillaene (BGC0001089, BGC0001090), Difficidin (BGC0000176), and Amylocyclicin (BGC0000616) were antimicrobial compounds encoded by gene clusters 6, 10, and 12, respectively. In addition, CH1 had gene clusters (2, 5, 6, 7) involved in the production of some antifungal compounds such as Surfactin (BGC0000433), Macrolactin H (BGC0001383, BGC0000181), Bacillomycin D (BGC0001090), iturin (BGC0001098), and fengycin (BGC0001095). Some active compounds detected in clusters 7and13 had dual antibacterial-antifungal activity, such as Pipastatin (BGC0000407) and Bacilysin (BGC0001184). It was also notable that Bacillibactin (BGC0001185) was a gene cluster encoding an iron carrier product.CH1 hindered the growth of plant-treating bacteria and fungi by competing for essential iron ions and thus played an important role in accelerating the acquisition of iron ions (Fe^3+^) from minerals and inter-root organic compounds^11^.

**Table 6:** Predicting genes and gene clusters encoding active metabolites in the genome of CH1.

Genes and gene clusters	Metabolites	Biological function	Coverage(%) *	Reference
*ybbR, glmM, glmS, potA, kdgR1*	andalusicin A/andalusicin B	Antibiotic (Fungi)	100	^12^
*YciC, yx01, xy02, YckC, YckD, YckE, Nin, NucA, HxlA, HxlB, hxlR, srfAA, srfAB, srfAC, srfAD, aat, ycxC, ycxD, sfp, yczE, yckI, yckJ*	surfactin	Antibiotic (Fungi)	99%	^13^
*bmmA, bmmB, bmmC, bmmD, bmmE, bmmF, bmmG, bmmH, bmmI* *pks2A, pks2B, pks2C, pks2D, pks2E, pks2F, pks2H, pks2I, pdhA*	Macrolactin H	Antibiotic (Fungi)	99%	^14^
*baeB, baeC, baeD, baeE, acpk, baeG, baeH, baeI, baeJ, baeL, baeM, baeN, baeR, baeS*	Bacillaene	Antibiotic (Bacteria), Induction of ISR	100%	^15^
*ynfF, XynD, bmyA, bmyB, bmyC, bmyD, yxjF, scoB, yxjC*	Bacillomycin D	Antibiotic (Fungi), Induction of ISR	97%	^13^
*ituA, ituB, ituD,*	iturin	Antibiotic (Fungi)	94%	^16^
*YngE, YngF, YngG, YngH, YngI, YngJ, YngK, YngL, fenA, fenB, fenC, fenD, fenE, DacC*	fengycin	Antibiotic (Fungi), Induction of ISR	100%	^17^
*pssE, pssD, pssC, pssB, pssA, yoeA*	Plipastatin	Antibiotics (Bacteria, Fungi)	100%	^18^
*difO, difN, difM, difA, difB, difC, difD, difE, difF, difG, difH, difI, difJ, difK, difL,*	Difficidin	Antibiotic (Bacteria)	98%	^13^
*DhbF, DhbA, DhbB, DhbC, DhbE*	Bacillibactin BGC0001185	Siderophore during iron deficiency in soil	100%	^19^
*ABS75250.1, ABS75251.1, ABS75252.1, ABS75253.1, ABS75254.1, ABS75255.1*	Amylocyclicin	Antibiotic (Bacteria), Induction of ISR	100%	^20^
*YwfG, BacE, BacD, BacC, BacB, BacA, YwfA,*	Bacilysin	Antibiotics (Bacteria, Fungi)	100%	^21^

*% of gene cluster similarity against MIBiG database.

#### Plant growth-promoting activity of CH1

In this study, The CH1 genome harbors numerous genes encoding proteins linked to plant growth-promoting activities, prominently including seven genes involved in the synthesis of IAA (Table 7). These genes are clustered within the tryptophan biosynthesis pathway, suggesting their participation in tryptophan-dependent IAA biosynthetic pathways. Moreover, some genes encode putative spermidine acetyltransferases and cytochrome P450 synthetases, which are predicted to produce spermidine and polyamines. Spermidine, a type of polyamine, has been shown to enhance plant growth and improve salt tolerance when accumulated within plants. Notably, the CH1 genome also comprises 12 genes that play a role in the development and regulation of biological membranes.

**Table 7:** Bioactivity-related genes in the CH1 genome.

Gene ID	Gene name	Protein coded by the gene	Reference
Genes associated with IAA production in the CH1 genome			
CH_1002040	*trpA*	tryptophan synthase alpha chain	^4^
CH_1002041	*trpB*	tryptophan synthase beta chain	^4^
CH_1002043	*trpC*	indole-3-glycerol phosphate synthase	^22^
CH_1002044	*trpD*	anthranilate phosphoribosyltransferase	^22^
CH_1000078	*trpE*	anthranilate synthase component I	^23^
CH_1002042	*trpF*	phosphoribosylanthranilate isomerase	^24^
CH_1000079	*trpG*	anthranilate synthase component II	^24^
Genes associated with spermidine production in the CH1 genome			
CH_1000698	*CYP102A2, CYP102A3*	cytochrome P450 / NADPH-cytochrome P450 reductase	^25^
CH_1003400	*speE*	spermidine synthase	^26^
CH_1003541	*msmX*	ABC-type spermidine transport systems	^26^
CH_1000551	*speG*	Spermidine acetyltransferas	^26^
Genes associated with biofilm formation, development and regulation in the CH1 genome			
CH_1002764	*iolU*	scyllo-inositol 2-dehydrogenase (NADP +)	^27^
CH_1000099	*cysE*	serine O-acetyltransferase	^28^
CH_1001603	*fliA*	RNA polymerase sigma factor for flagellar operon FliA	^29^
CH_1001694	*hfq*	host factor-I protein	^30^
CH_1001999	*crr*	sugar PTS system EIIA component	^31^
CH_1002045	*trpE*	anthranilate synthase component I	^31^
CH_1002732	*luxS*	S-ribosylhomocysteine lyase	^32^
CH_1003089	*rpoN*	RNA polymerase sigma-54 factor	^33^
CH_1003193	*csrA*	carbon storage regulator	^34^
CH_1003199	*flgM*	negative regulator of flagellin synthesis FlgM	^35^
CH_1003222	*wecB*	UDP-N-acetylglucosamine 2-epimerase (non-hydrolysing)	^36^
CH_1003229	*tagA, tarA*	N-acetylglucosaminyldiphosphoundecaprenol N-acetyl-beta-D-mannosaminyltransferase	^37^

#### CH1 is involved in symbiotic nitrogen fixation and tumorigenesis

The CH1 genome contains genes predicted to produce arabinogalactanase, pectate lyase, and xylanase, as well as the *guaB* gene encoding an IMP dehydrogenase, which is predicted to be involved in the nodulation process in conjunction with rhizobia. In addition, three genes *(cysK*, *cysE*, and *ilvE*) were detected in the CH1 genome and are predicted to be involved in cysteine biosynthesis. An enhanced plant nitrogen fixation ability can promote plant growth and development.

## Discussion

NCBI's genomic phylogenetic analysis and 16S rDNA homology analysis reveal that CH1 exhibits a high degree of homology with KS04AU, YL2021, YYC, and LZN01, originating from diverse sources. The homology analysis based on 16S rDNA sequencing is a commonly employed taxonomic method for bacteria^25^. Among the six *Bacillus velezensis* strains analyzed, CH1 shows the closest relationship to YYC based on 16S rDNA homology, which is further supported by the Average Nucleotide Identity (ANI) analysis. *Bacillus velezensis* YYC was isolated from tomato rhizosphere soil. Given the aforementioned findings, there appears to be no significant correlation between the strains and their host sources.

In this study, biochemical characterizations and a comparative genome analysis were performed to survey the potential biology characteristics of CH1. *B. velezensis* was found to be nitrogen-fixing and capable of forming biofilms^6^. It has been shown that the antagonistic activity and biocontrol effects of *Bacillus* are related to its ability to form biofilms^38,39^. *B. velezensis* was also shown to produce IAA to promote plant growth^4,5^. It was also confirmed in this study that CH1 produced IAA. Before this study, there was little research on whether *B. velezensis* encoded an ACC deaminase, produced an iron carrier, or solubilized phosphate. ACC deaminase directly promotes plant growth and indirectly inhibits plant diseases^40^. Recently, ACC deaminase has been discovered in various Gram-negative bacteria, fungi, endophytes, and rhizobia^39^. However, the expression level of ACC deaminase varies among different organisms. Currently, the entire genetic composition and functions of ACC deaminase genes have only been described in a limited number of bacterial species, while most of the relevant bacterial genes are unknown^41^. Early studies also found that some genes encoding ACC deaminase activity also encode D-cysteine desulfhydrase, suggesting that changes in specific gene loci could potentially be attributed to D-cysteine desulfhydrase^42^. Reports have documented that *B. velezensis* possesses ACC deaminase activity^43^. In this study, we also observed that CH1 exhibits a certain level of ACC deaminase activity, yet a complete gene (acdS) related to this activity was not identified in the CH1 genome. Instead, we found a homocysteine desulfhydrase gene, which may play a significant role in the expression of ACC deaminase activity in *B. velezensis*.

IAA biosynthesis involves both tryptophan-dependent and tryptophan-independent pathways, with the former being the most dominant and common pathway^44,45^. In addition, iron carriers make iron useful as an enzyme cofactor for the shuffling of electrons, therefore enabling transition metals to be involved in many basic biological processes, such as respiration, photosynthesis, and nitrogen fixation^46^. Most of the inorganic phosphorus salts applied as fertilizers in soil consolidate quickly after application and become unavailable. Inoculation of seeds or soil with bacteria that increase phosphorous could improve the solubility of soil phosphorus and exert an equivalent effect of phosphorus fertilization^47^. However, although CH1 bacteria grew in the NBRIP solid medium, there was no transparent trend compared with positive control, which confirmed that CH1 did not solubilize phosphate.

CH1 demonstrated significantly higher inhibition than the negative control group (PBS) on *Bipolaris sorokiniana, Powdery mildew, and Tilletia controversa* in this study. *Bipolaris sorokiniana* is a worldwide plant pathogenic fungus, which can infect the underground and above-ground parts of wheat and cause wheat root rotis. *Tilletia controversa* is a fungal disease characterized by a black whiplash (black spike) growing from the apex of the stem*. Tilletia controversa* causes smut in wheat*. Powdery mildew occurs* in leaves, stems, stalks, buds, and petals. The affected area turns gray, the leaves shrink and become smaller when the damage is severe, the tender ends are twisted and deformed, and the flower buds do not open. These results confirmed that *B. velezensisto* inhibited the growth of the main pathogenic bacteria of whea^20,29,48,49^. The inhibition by CH1 of these pathogens might be through the production of metabolites. The secondary metabolites might be important for plant growth and defense^29^; volatile organic compounds of *B. velezensis* were found to be involved in biocontrol^49^. Moreover, antimicrobial active substances were found in *B. velezensis*^20,49^. Antibacterial compounds not only inhibit the growth of plant pathogens and fungi but also stimulate the induction of systemic resistance in plant^50^. The largest proportion of annotations in the COG database was metabolism (44.79%), including biosynthesis, transport, and catabolism of secondary metabolites. The enrichment of these functions was strong evidence of the antimicrobial properties of strain CH1. The secondary metabolites are potentially associated with participation in the cluster production of antimicrobials, including bacillin, surfactin, metallatin, arosetin, bacillin, dysgentin, and terpenes. However, clusters that predict the production of antipeptides are not typical in other bacillus. Antipeptides are ribosomally synthesized peptides that undergo extensive post-translational modifications and are antimicrobial compounds produced by gram-positive bacteria^51^. Biological nitrogen fixation is an important factor in promoting plant growth^52^.

*B. velezensis* is able to secrete a rich variety of secondary metabolites, which is an important prerequisite for its beneficial effects on plants such as growth promotion and disease prevention^53^. Wu et al. found that the genome of *B. velezensis* NAU-B3 contains eight genes clusters coding for secondary metabolites with known functions, such as bacillomycin D, bacilysin, and bacillaene, and one genes cluster with unknown functions, which are synthesized in the NPRS, PKS, and PRS pathways, and have the effects of inhibition of bacteria and fungi, formation of biofilm, and accumulation of ferric iron ions, etc^54^. In the present study, we found that the genome of CH1 is rich in gene clusters, which are effective for promoting the growth of the plant and defense. Thirteen gene clusters were identified in the CH1 genome, which were responsible for the synthesis of 15 secondary metabolites, including those encoding andalusicin, macrolactin, bacillaene, fengycin, difficidin, and bacillothiazol, bacillbactin, bacilysin, etc. The gene clusters encoding the synthesis of eight inhibitory products were identical to the identified gene clusters, and the similarity between the surfactin synthesis gene cluster and the identified gene clusters was as high as 91%. In the surfactin cluster, the *xy02* gene is unique to CH1 in the surface protein cluster and is a derivative of CA4P. Furthermore, *xy02* exhibited a certain growth inhibition effect on human non-small cell lung cancer NCI-H460 cells^55^. However, it was also shown that although CH1 had a large number of specific genes distributed in the genome, the function of most of the specific genes was unknown. Further investigation will help to understand the molecular mechanisms of the prebiotic properties of strain CH1. These secondary metabolites have been reported to antagonize phytopathogenic bacteria, fungi, and viruses, induce plant disease resistance, accumulate iron ions, and form biofilms, which suggests that the bioprophylactic bacterium CH1 has a good application value. In addition, three unknown clusters of secondary metabolites synthesizing genes were predicted from the genome of CH1, which will provide a basis for exploring the potential of CH1 for bioprevention and discovering novel bacterial inhibitors in the future.

A number of genes that may be related to plant growth-promoting activity have been identified in the CH1 genome. The CH1 genome has *trpA*, *trpB*, *trpC*, *trpD*, *trpE*, *trpF*, and *trpG* genes involved in the synthesis of IAA from indole-3-acetonitrile^24^. Therefore, we suggest that CH1 contains genes functionally related to growth hormone synthesis and plays an important role in promoting plant growth and development^56^. Moreover, *CYP102A2/CYP102A3* are genes encoding cytochrome P450, while *speE*, *msmX*, and *speG* are genes encoding enzymes involved in spermidine and polyamine synthesis, which are believed to play a role in plant development and growth promotion through participation in the synthesis of active metabolites^57^. The development of biofilms in the strain promotes plant growth and protects the plant from infecting microorganisms through the secretion of antimicrobial compounds^28^. CH1 has several genes involved in biofilm development. It has been shown that many genes are involved in the synthesis of Fengycin, Bacillibactin, while Bacilysin is upregulated during biofilm formation^34^. We expected that CH1 might promote rhizoma formation because some genes associated with rhizoma formation were detected in the CH1 genome, and these genes were predicted to produce arabinogalactanase, pectate lyase, and xylanase, which may weaken the root hair cell walls, facilitate bacterial invasion, and contribute to form rhizomes^58^. Another study shows that the* guaB* gene encoding guanine is efficiently expressed during the early stages of rhizoma formation^59^.

## Conclusion

In the study, CH1 was isolated, purified, and identified from high-quality oat varieties. The physiological indexes of CH1 and the whole genome of CH1 were determined to verify the effect of CH1 on promoting the growth of oat and inhibiting the main diseases of oat. It was confirmed that CH1 might be a potential biofertilizer for promoting oat growth and disease resistance.

## Materials and methods

### Isolation and purification of the strain

The oat plants used in this experiment were Baiyan No.2, supplied by Ulanqab Agricultural and Animal Husbandry Academy, and Inner Mongolia Academy of Agriculture and Animal Husbandry Sciences in China, which possess a strong ability to inhibit disease. The oat plants were rinsed well with tap water and then rinsed five times with sterile PBS. They were cut into smaller pieces with sterile scissors, homogenized in a small homogenizer, and drained into a paste. The plant tissue paste was eluted with PBS into a sterilized glass bead flask and fully oscillated. Cells from plant tissues were cultured in trypticase soy broth (TSB) solid medium using multiple dilutions and the pour culture method. Then, the plates were cultured in an anaerobic incubator and thermostatic incubator at 37°C for 24–36 h. After the bacteria grew, a single colony was selected that had a white color, wrinkled surface, dry texture, and filamentous mucus inside the colonies, and the bacteria were positive upon Gram staining. The bacteria were marked until the colonies were single and typical. This study was approved by the Plant Protection Association of Inner Mongolia Autonomous Region and the Professional Committee of Jining Normal University of Inner Mongolia Autonomous Region (Ulanqab, China; Approval No. 20221012), and complied with the IUCN Policy Statement on Research Involving Species at Risk of Extinction and the Convention on the Trade in Endangered Species of Wild Fauna and Flora.

### Identification of the strain

The physiological and biochemical characteristics of the purified strain were identified using an HBI *Bacillus* Biochemical Identification strip (GB) (Qingdao Hope Bio-Technology Co., LTD., Qingdao, China), and 16s RNA sequence analysis based on universal primers (27F and 1492R) was used to confirm the strain. The physiological and biochemical experiment process is detailed as follows: The bacteria were cultured in an anaerobic incubator, a constant temperature incubator, and an anaerobic bag in a constant temperature incubator to test whether the bacteria grew in an anaerobic state, an aerobic state, or a facultative anaerobic state. The experimental bacteria were inoculated with a straight needle in a semi-solid medium and cultured in an anaerobic incubator, and their motility could be observed through the optical eye. If the culture grew on the puncture line of the inoculation and the edge was very clear, it indicated that the test bacteria had no motility; if the growth of the culture spread from the puncture line to the periphery in a cloud shape, and the edge was blurred into a cloud shape, it indicated that the bacteria had motility. Take a ring of culture coated on a clean slide, and then add a drop of 3–15% hydrogen peroxide onto it, if there is a bubble, it is a positive reaction, if there is no bubble is a negative reaction. The culture was inoculated in a gelatin medium for culture, and the results were observed by low-temperature treatment. The inoculated and uninoculated pairs were placed in the refrigerator for solidification and then removed, and their liquefaction status was compared. For example, when the inoculated tubes were solidified, the results were positive for liquefaction and negative for coagulation or liquefaction at the same time. The culture was mixed with 40% NaOH and 1 mg creatine at room temperature for 30 to 60 minutes, and the red reaction was positive and unchanged into a negative reaction. The bacteria were inoculated in a sugar fermentation medium, and if the result turned yellow, it indicated that the carbohydrate acid production was positive, and if it did not change color, it was negative. Each experiment was repeated three times. Then, the amplified products were sequenced, and phylogenetic analysis was performed with the software MEGA version 6.0.

### CH1 biochemical features

The biochemical features of strain CH1 were detected by a series of biochemical tests, including assays for ACC deaminase, nitrogen, solubility of phosphate, iron carrier formation, IAA production, biofilm formation, and antibacterial activity. All biochemical tests were according to standard experimental procedures and followed the manufacturer’s instructions. In addition, the Oxford cup method was used for detecting antibacterial activity^60^. The diameter of the bacteriostatic zone was measured accurately with vernier calipers to determine the bacteriostatic ability.

### Whole-genome sequencing and functional annotation

After activation of strain CH1, bacteria were collected by centrifugation. Following the manufacturer's instructions, the genomic DNA of the strain was extracted using the bacterial DNA extraction kit (Omega Bio-Tek, USA). Subsequently, the purified DNA samples underwent quality control. The genomic DNA was quantified using the TBS-380 fluorometer (Turner BioSystems Inc., Sunnyvale, CA). Illumina libraries were constructed using the TruSeq™ Nano DNA Sample Prep Kit (Illumina, USA) and sequenced on the Illumina novaseq6000 platform. For PacBio sequencing, 20k insert whole-genome shotgun libraries were generated and sequenced using standard methods on the PacBio RS instrument. Illumina sequencing data were employed for correcting PacBio sequencing data and simultaneously assessing the complexity of the genome. Following genome assembly and result evaluation, genome composition analysis was conducted. Gene prediction for the strain CH1 genome was performed using GeneMarkS (Version 4.17, http://topaz.gatech.edu/GeneMark/) software. The prediction of rRNA, tRNA, and other ncRNA contained in the genome was accomplished using RNAmmer (version 1.2, http://www.cbs.dtu.dk/services/RNAmmer/), tRNAscan-SE (version 2.0.4, http://lowelab.ucsc.edu/tRNAscan-SE), and Infernal (version 1.1.14) softwar^61–63^. The eggNOG (http://www.ncbi.nlm.nih.gov/COG/) database was applied to the CH1 genome-wide data for orthologous (COG) functional annotation. The SwissProt (http://uniprot.org) database was used to annotate growth-promoting genes in CH1.

### Comparative genomics analysis

Based on the whole genome sequence of strain CH1, the whole genome sequences and annotations of 201 *B. velezensis* strains isolated from different hosts and environments were obtained from the GenBank genome database (https://www.ncbi.nlm.nih.gov/genome/). Genome phylogenomic analysis of 206 *B. velezensis* strains was performed using REALPHY (http://realphy.unibas.ch/realphy). Orthologous Average Nucleotide Identity Tool (v0.93.1) was used to calculate OrthoANI and type the CH1 genom^64^. Digital DNA-DNA hybridization (dDDH) analysis was performed by Genome-to-Genome Distance Calculator (GGDC v 3.0, https://ggdc.dsmz.de/ggdc.php)^65^. Utilizing the MEGA version 6.0 software, a phylogenetic tree was constructed based on the full-length 16S rRNA gene sequences of six closely related *Bacillus velezensis* strains. A comparative whole-genome analysis was further conducted, including strains closely affiliated to CH1 as well as *Bacillus* reference strains (KS04AU^66^, YYC^67^, FZB42^17^, DSM7^68^, and 168^69^). The complete genome sequences of strains KS04AU, YYC, FZB42, DSM 7, and 168 were downloaded from NCBI Genome and then analyzed together using comparative genomics with the complete genome sequences of CH1. CD-HIT 4.6.6 (http://cd-hit.org) software was used for cluster analysis, core genes and specific genes of the genome were determined^70^, and the number of common and specific gene petals was constructed. A phylogenetic tree was constructed using PhyML (Maximum Likelihood, version 3.0, http://www.atgc-montpellier.fr/phyml/) software. AntiSMASH version 7.1.0 software (https://antismash.secondarymetabolites.org) was used to predict the scope of secondary metabolite synthesis^71^. Meanwhile, the predicted gene clusters of secondary metabolite synthesis were compared and analyzed. Using antiSMASH 7.1.0 on the default settings, the structure of secondary metabolism was detected against MIBiG.

### Supplementary Information

Supplementary Information.

## Data Availability

The datasets and accession to cite for these SRA data: PRJNA951273 of this study in NCBI. All of the data generated or analyzed during this study are included in this published article, and the supplementary information files will be freely available to any scientist wishing to use them for non-commercial purposes upon request via e-mail.
