# Supplementary material for: A Bacillus velezensis strain isolated from oats with disease-preventing and growth-promoting properties
Source: Sci Rep. 2024 Jun 5;14:12950. doi: 10.1038/s41598-024-63756-8 (PMC11153497; doi:10.1038/s41598-024-63756-8)
Supplement: Supplementary file 1 — Supplementary Information. [file 41598_2024_63756_MOESM1_ESM.docx]

**A *Bacillus velezensis* strain isolated from oats with disease-preventing and growth-promoting properties**

Chao Cheng, Shaofeng Su, Suling Bo, Chengzhong Zheng, Chunfang Liu, Linchong Zhang, Songhe Xu, Xiaoyun Wang, Pengfei Gao, Kongxi Fan, Yiwei He, Di Zhou, Yanchun Gong, Gang Zhong, Zhiguo Liu

**1** **SUPPLEMENTARY** **FIGURES**


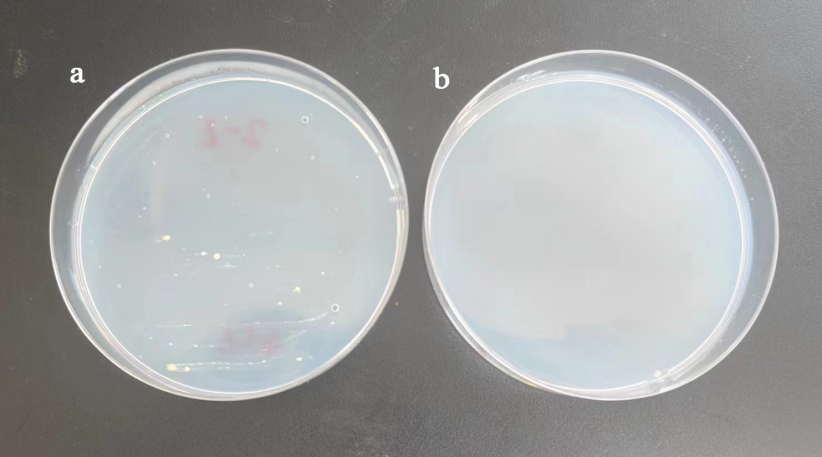


**Figure** **S1.** The results of ACC decomposition of CH1. (a) the experimental group; (b) the control group.


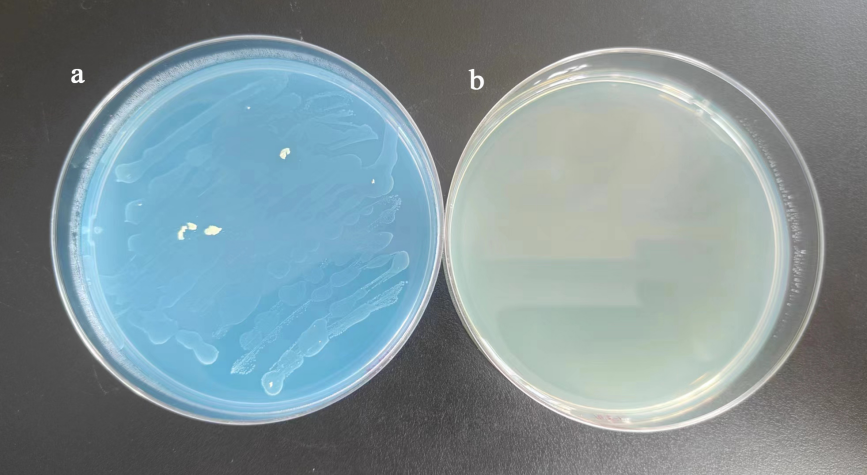


**Figure** **S2.** The results of nitrogen fixation function of CH1. (a) the experimental group; (b) the control group.


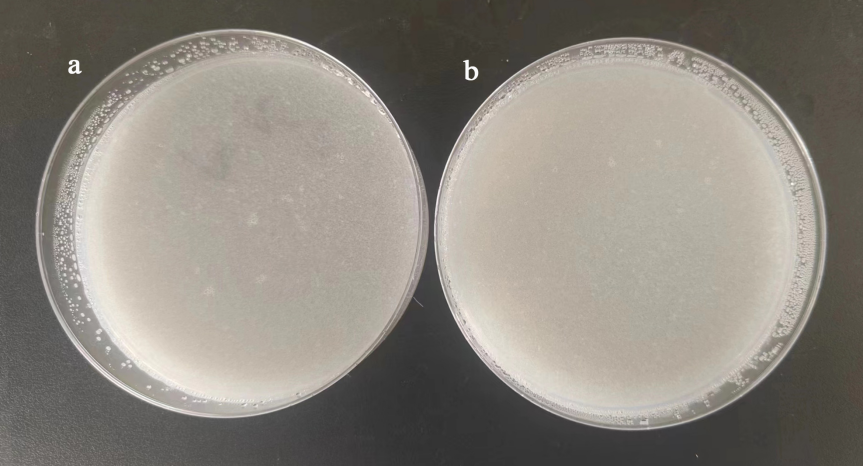


**Figure** **S3.** The results of the phosphate solubility function of CH1. (a) the experimental group; (b) the control group.


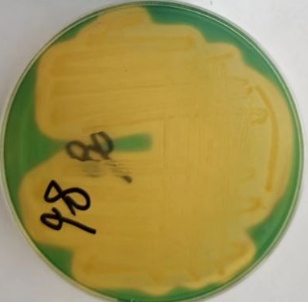


**Figure** **S4.** The results of CH1 producing ferricarriers.

**
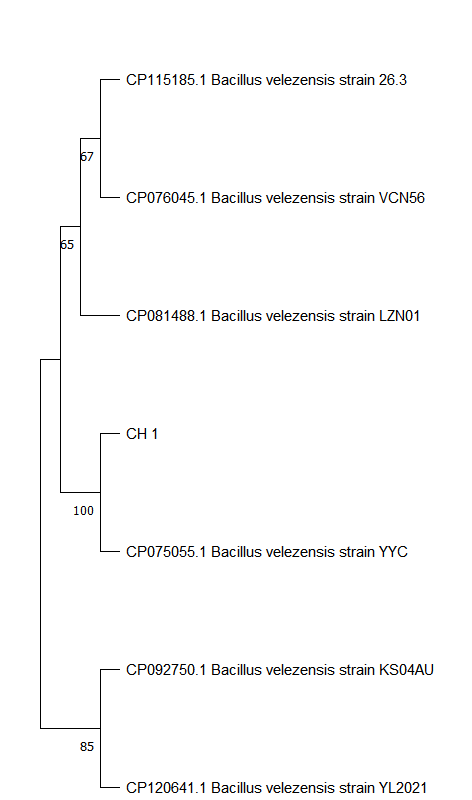
**

**Figure S5.** Phylogenetic tree map based on 16S rRNA of *B. velezensis*.

**Figure 1a**


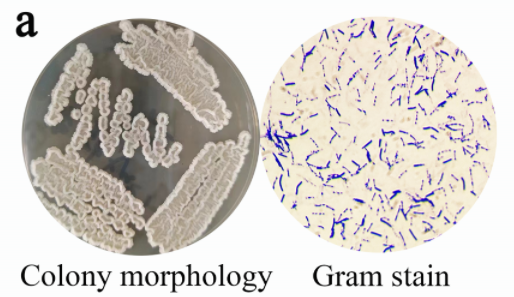


Gram stain

Colony morphology


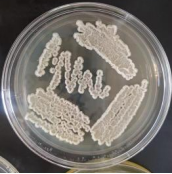

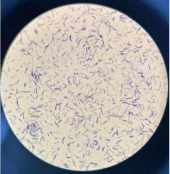


**Figure 1b**


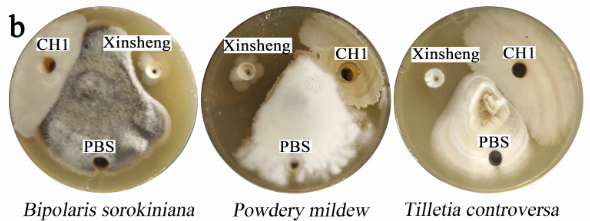


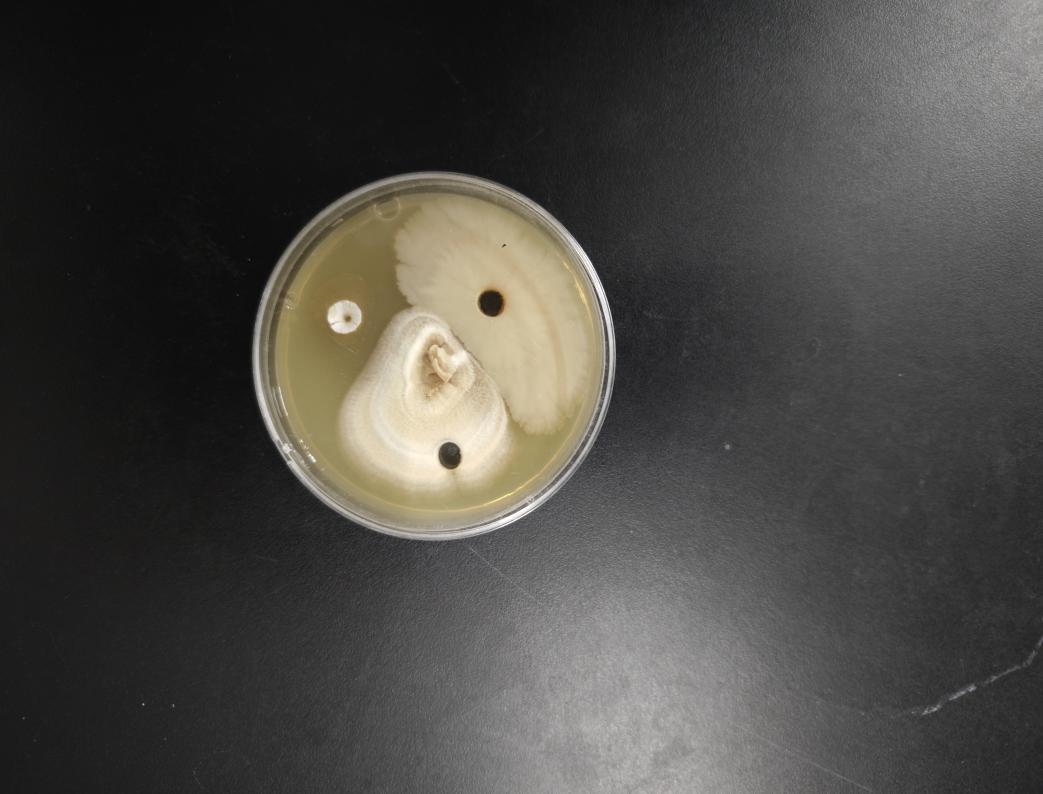

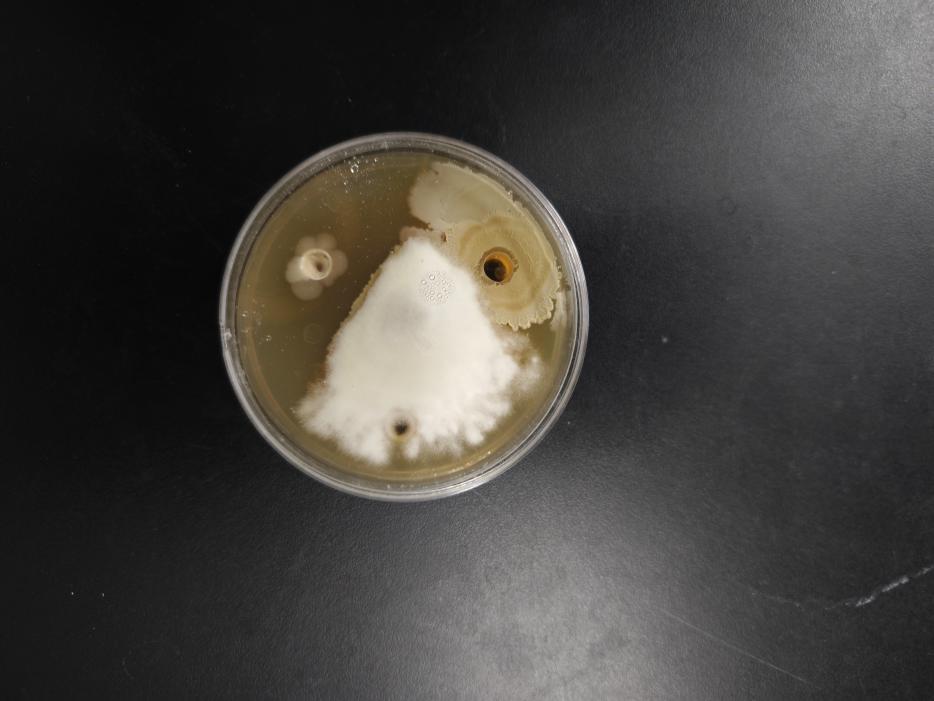

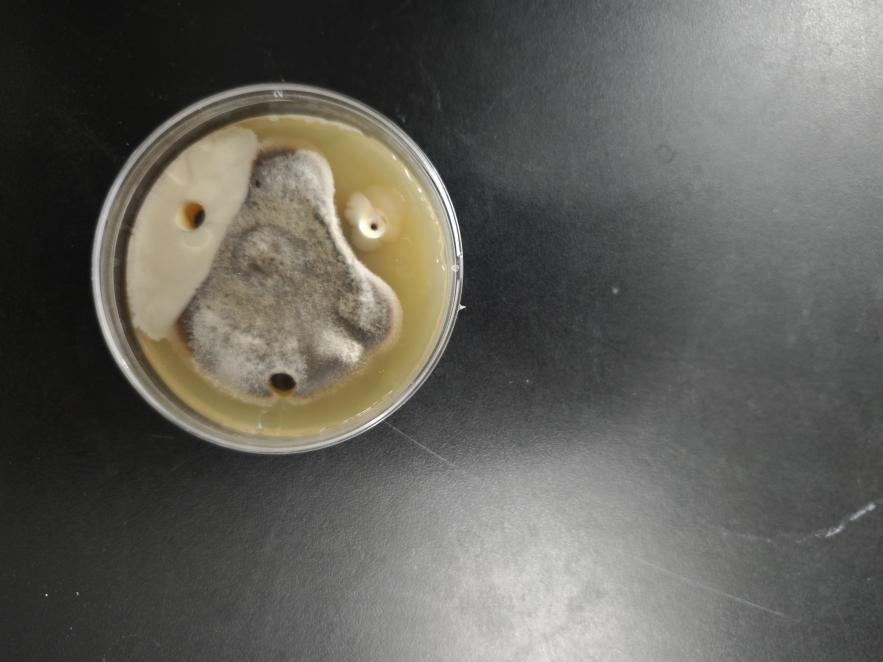


*Tilletia controversa*

*Powdery mildew*

*Bipolaris sorokiniana*

**Figure S6.** The original images of the culture plates.

**2** **SUPPLEMENTARY** **TABLES**

| Species | Strain | Accession | OrthoANI | DDH | Model C.I. | Distance |
| --- | --- | --- | --- | --- | --- | --- |
| 1. *velezensis* | KS04AU | GCA_022488425.1 | 99.3371 | 95.70% | [93.5 - 97.1%] | 0.0471 |
| *B. velezensis* | YYC | GCA_019163475.1 | 99.3418 | 97.90% | [96.6 - 98.8%] | 0.0295 |
| *B. velezensis* | LZN01 | GCA_019754015.1 | 99.3338 | 97.90% | [96.6 - 98.8%] | 0.0295 |
| *B. velezensis* | 26.3 | GCA_027625395.1 | 99.3402 | 97.90% | [96.6 - 98.8%] | 0.0294 |
| *B. velezensis* | VCN56 | GCA_018629015.1 | 99.3144 | 97.90% | [96.6 - 98.8%] | 0.0294 |
| *B. velezensis* | YL2021 | GCA_029537355.1 | 99.312 | 95.70% | [93.6 - 97.2%] | 0.0464 |
| *B. velezensis* | FZB42 | GCA_000015785.2 | 98.9177 | 96.10% | [94 - 97.4%] | 0.0442 |
| *B. amyloliquefaciens* | DSM 7 | GCA_000196735.1 | 94.1599 | 81.90% | [78 - 85.2%] | 0.1228 |
| *B. subtilis* | 168 | GCA_000009045.1 | 76.8987 | 33.70% | [30.4 - 37.3%] | 0.4621 |

**Table S1.** OrthoANI and dDDH calculation of the strain CH1.

| Compound | Gene | Presence (+) or absence (−) | | | | | | Functions or product |
| --- | --- | --- | --- | --- | --- | --- | --- | --- |
|  |  | CH1 | KS04AU | YYC | FZB42 | DSM7 | 168 |  |
| Surfactin | *YciC* | + | + | + | + | + | + | YciC_protein |
|  | *yx01* | + | + | + | + | + | - | Yx01 protein |
|  | ***xy02*** | + | - | - | - | - | - | Xy02 protein |
|  | *YckC* | + | + | + | + | + | + | YckC protein |
|  | *YckD* | + | + | + | + | + | + | YckD protein |
|  | *YckE* | + | + | + | + | + | + | YckE protein |
|  | *Nin* | + | + | + | + | + | + | Nin |
|  | *NucA* | + | + | + | + | + | + | NucA |
|  | *HxlA* | + | + | + | + | + | + | HxlA protein |
|  | *HxlB* | + | + | + | + | + | + | HxlB protein |
|  | *hxlR* | + | + | + | + | + | + | transcriptional_regulato |
|  | *srfAA* | + | + | + | + | + | + | Surfactin synthetase A, scaffold biosynthesis |
|  | *srfAB* | + | + | + | + | + | + | Surfactin synthetase B, scaffold biosynthesis |
|  | *srfAC* | + | + | + | + | + | + | Surfactin synthetase C, scaffold biosynthesis |
|  | *srfAD* | + | + | + | + | + | + | Surfactin synthetase D |
|  | *aat* | + | + | + | + | + |  | Amino transferase |
|  | *ycxC* | + | + | + | + | - | + | Transporter |
|  | *ycxD* | + | + | + | + | - | + | Transcriptional regulator containing an amino transferase domain |
|  | *sfp* | + | + | + | + | + | + | phosphopantetheinyl transferase involved in nonribosomal synthesis |
|  | *yczE* | + | + | + | + | + | + | integral membrane protein involved in nonribosomal synthesis |
|  | *yckI* | + | + | + | + | + | + | YckI protein |
|  | *yckJ* | + | + | + | + | + | + | YckJ protein |
| Fengycin | *YnfF* | + | + | + | + | + | + |  |
|  | *XynD* | + | + | + | + | + | + |  |
|  | *bmyC* | + | + | + | + | + | - | bacillomycin D synthetase C, Scaffold biosynthesis |
|  | *bmyB* | + | + | + | + | + | - | bacillomycin D synthetase B, Scaffold biosynthesis, Scaffold biosynthesis |
|  | *bmyA* | + | + | + | + | + | - | bacillomycin D synthetase A, Scaffold biosynthesis |
|  | *ituA* | + | + | + | + | + | - | iturin A synthetase A |
|  | *ituB* | + | + | + | + | + | - | Iturin A synthetase B |
|  | *ituD* | + | + | + | + | + | - | putative malonyl-CoA transacylase |
|  | *bmyD* | + | + | + | + | + | - | malonyl-CoA transacylase, Scaffold biosynthesis |
|  | *YxjF* | + | + | + | + | + | - |  |
|  | *scoB* | + | + | + | + | + | - |  |
|  | *scoA* | + | + | + | + | + | - |  |
|  | *YxjC* | + | + | + | + | - | - |  |
|  | *YngE* | + | + | + | + | + | + |  |
|  | *YngF* | + | + | + | + | + | + |  |
|  | *YngG* | + | + | + | + | + | + |  |
|  | *YngH* | + | + | + | + | + | + |  |
|  | *YngI* | + | + | + | + | + | + |  |
|  | *YngJ* | + | + | + | + | + | + |  |
|  | *YngK* | + | + | + | + | + | + |  |
|  | *YngL* | + | + | + | + | + | + |  |
|  | *yoeA* | + | + | + | + | + | + | Putative efflux transporter |
|  | *DacC* | + | + | + | + | + | + |  |
|  | *fenA* | + | + | + | + | + | + | Fengycin synthetase A, scaffold biosynthesis |
|  | *fenB* | + | + | + | + | - | + | Fengycin synthetase B, scaffold biosynthesis |
|  | *fenC* | + | + | + | + | - | + | Fengycin synthetase C, scaffold biosynthesis |
|  | *fenD* | + | + | + | + | - | + | Fengycin synthetase D, scaffold biosynthesis |
|  | *fenE* | + | + | + | + | + | + | Fengycin synthetase E, scaffold biosynthesis |

**Table S2.** Comparative analysis of antimicrobial gene clusters in secondary metabolites.
